# Antibiotics versus no treatment for asymptomatic bacteriuria in residents of aged care facilities: a systematic review and meta-analysis

**DOI:** 10.3399/BJGP.2022.0059

**Published:** 2022-08-09

**Authors:** Natalia Krzyzaniak, Connor Forbes, Justin Clark, Anna Mae Scott, Chris Del Mar, Mina Bakhit

**Affiliations:** Institute for Evidence-Based Healthcare, Faculty of Health Sciences and Medicine, Bond University, Gold Coast, Queensland, Australia.; Institute for Evidence-Based Healthcare, Faculty of Health Sciences and Medicine, Bond University, Gold Coast, Queensland, Australia.; Institute for Evidence-Based Healthcare, Faculty of Health Sciences and Medicine, Bond University, Gold Coast, Queensland, Australia.; Institute for Evidence-Based Healthcare, Faculty of Health Sciences and Medicine, Bond University, Gold Coast, Queensland, Australia.; Institute for Evidence-Based Healthcare, Faculty of Health Sciences and Medicine, Bond University, Gold Coast, Queensland, Australia.; Institute for Evidence-Based Healthcare, Faculty of Health Sciences and Medicine, Bond University, Gold Coast, Queensland, Australia.

**Keywords:** antibiotics, asymptomatic bacteriuria, bacteriuria, meta-analysis, residential aged care facilities, systematic review

## Abstract

**Background:**

Asymptomatic bacteriuria (ASB) is common among residents of residential aged care facilities (RACFs). However, differentiating between an established urinary tract infection and ASB in older adults is difficult. As a result, the overuse of dipstick urinalysis, as well as the subsequent initiation of antibiotics, is common in RACFs.

**Aim:**

To find, appraise, and synthesise studies that reported the effectiveness, harms, and adverse events associated with antibiotic treatment for older patients with ASB residing in RACFs.

**Design and setting:**

A systematic review using standard Cochrane methods of RACF residents with ASB using antibiotics against placebo, or no treatment.

**Method:**

Three electronic databases (PubMed, EMBASE, and CENTRAL), clinical trial registries, and forward–backward reference checks of included studies were searched.

**Results:**

Nine randomised controlled trials, comprising 1391 participants were included; two of which used a placebo comparator, and the remaining seven used no therapy control groups. There was a relatively small number of studies assessed per outcome and an overall moderate risk of bias. Outcomes related to mortality, development of ASB, and complications were comparable between the two groups. Antibiotic therapy was associated with a higher number of adverse effects (four studies; 303 participants; risk ratio [RR] 5.62, 95% confidence interval [CI] = 1.07 to 29.55, *P* = 0.04) and bacteriological cure (nine studies; 888 participants; RR 1.89, 95% CI = 1.08 to 3.32, *P* = 0.03).

**Conclusion:**

Overall, although antibiotic treatment was associated with bacteriological cure, it was also associated with significantly more adverse effects. The harms and lack of clinical benefit of antibiotic use for older patients in RACFs may outweigh the benefits.

## INTRODUCTION

The high use and misuse of antibiotics within residential aged care facilities (RACFs) is well recognised as a significant concern within healthcare systems.^1^ The non-specific symptoms associated with infection in the older patient population, as well as the fear of patient deterioration, often leads to the initiation of early antibiotic therapy as a safety net.^2^^,^^3^ In Australian RACFs, recurring antibiotic use issues have been consistently identified since 2016.^4^ These issues include: high rates of antibiotic use for residents who do not meet the criteria for infection, use of antibiotics prophylactically for urinary tract infections (UTIs) and for a duration lasting longer than 6 months, high use of broad-spectrum antibiotics, no adherence to national guideline recommendations, and poor-quality documentation around antibiotic prescriptions.^4^

Asymptomatic bacteriuria (ASB) in particular is common among residents of RACFs, with estimated prevalence rates in females of 25%–50% and 15%–40% in males.^5^^–^7 High rates of urinary retention, urinary incontinence, frailty, increased use of invasive devices, immobility, comorbidities, and decreased immunity, as well as increased exposure to organisms (resident– resident, resident–visitor, and resident–carer contact), increase potential infection risk among residents.^2^ Differentiating between an established UTI and ASB in older adults is difficult.^8^^,^^9^ Patients with chronic symptoms or cognitive impairments are often unable to recognise or communicate the symptoms of a UTI and this coupled with non-specific presentation of symptoms renders this condition particularly challenging for clinicians to diagnose.^8^^,^^10^ As a result, despite poor-quality evidence supporting the use of dipstick tests in patients >65 years of age, these are commonly overused in RACFs and lead to the subsequent initiation of antibiotics.^11^

A Cochrane systematic review evaluated the evidence of the safety and effectiveness of antibiotics prescribed for ASB in the adult population (patients >18 years of age) and in any healthcare setting.^12^ The authors found no clinical benefit in treating ASB, with no significant differences between antibiotic therapy and no therapy in the development of symptomatic UTI, complications, or death. However, antibiotics were associated with significantly more adverse events and it is not clear if the results would be applicable to RACF residents,^12^ thus highlighting the need for the present review, which specifically focuses on older adults in long-term care facilities.

Older patients are at particular risk of adverse drug reactions as a result of polypharmacy as well as changes in organ sensitivity and pharmacokinetics.^13^ Systematic reviews of antibiotic-associated harms are generally unavailable for older patients in long-term care facilities; however, different studies have reported on antibiotic-associated harms for this population.^14^^–^16

**Table:** How this fits in

Asymptomatic bacteriuria (ASB) is often treated with antibiotics, contributing to the global burden of antibiotic resistance. Current evidence suggests no clinical benefit in treating ASB, with no significant differences between antibiotic therapy and no therapy in the development of symptomatic urinary tract infections, complications, or death. However, it is not clear if the results would be applicable to residents of residential aged care facilities. This study found that, although antibiotic therapy was associated with bacteriological cure, it was also associated with significantly more adverse effects. The harms and lack of clinical benefit of antibiotic use for older patients in residential aged care facilities may outweigh the benefits.

This systematic review focuses on assessing the effectiveness and harms of antibiotics treatment versus no antibiotics for residents of RACFs who have ASB.

## METHOD

The aim of the study was to find, appraise, and synthesise studies that reported the effectiveness, harms, and adverse events associated with antibiotics treatment for older patients with ASB residing in RACFs. The protocol for this systematic review was prospectively developed and registered at the Center for Open Science on 3 December 2021 (https://osf.io/f8uka). This systematic review followed the 2-week systematic review process^17^ and is reported following the Preferred Reporting Items for Systematic Reviews and Meta-Analyses checklist (PRISMA 2020).^18^

### Inclusion and exclusion criteria

#### Study design

Randomised controlled trials (RCTs), non-RCTs, and observational studies with a comparator group (cohort or case– control studies) were included. Case reports, case series, letters to the editor, before and after, interrupted time series, cross-sectional design studies, qualitative studies, and reviews (for example, systematic, literature, narrative, and meta-analyses) were excluded.

#### Population/participants

Studies were included if their populations of interest comprised individuals residing in RACFs, who were diagnosed with an ASB or bacteriuria. However, studies involving older patients in hospitals or those residing in their own homes, or who were attending GP or community health clinics were excluded.

#### Intervention

The intervention was therapeutic or prophylactic antibiotic treatment of any type, dose, duration, or administered by any route of delivery (that is, oral, topical, intravenous, and intramuscular). The use of concomitant medications was permitted, if also given to the comparator group.

#### Comparators

Studies with a comparator involving placebo, no prescribing, delayed prescribing, or withheld prescribing of antibiotics were included.

#### Outcomes (primary and secondary)

The primary outcomes were development of symptomatic UTI, any cause of mortality, and adverse effects of antibiotic use. Secondary outcomes included antibiotic resistance, disease complications, and bacteriological cure or recurrence.

### Information sources and search strategy

Electronic databases, including PubMed (Medline), EMBASE, and CENTRAL via the Cochrane Library, were searched for potentially relevant primary studies from inception until November 2021. The search string was designed in PubMed, and translated for use in other databases using the Polyglot Search Translator.^17^^,^^19^

The following components were included in the search string: MeSH terms (that is, anti-infective agents, infections, homes for the aged, and adverse effects) or other subject terms, synonyms, and search filters. Search strings were constructed and run by a Cochrane information specialist. The complete list of search strings for all databases are provided in Supplementary Box S1.

In addition, a backwards and forwards citation search of the included studies was undertaken using Scopus on all of the included studies identified in the database searches, to identify any further relevant studies.

Ongoing trials in clinical registries were searched for via Cochrane CENTRAL, which contains the World Health Organization International Clinical Trials Registry Platform and clinicaltrials.gov.

No restrictions by language or publication date were imposed. Publications that were published in full were included; publications available as an abstract only (for example, a conference abstract) were included only if they had a clinical trial registry record, or other public report, with the additional information required for inclusion. Publications available as an abstract only (for example, a conference abstract) with no additional information available were excluded.

### Screening and data extraction

**Figure 1.**
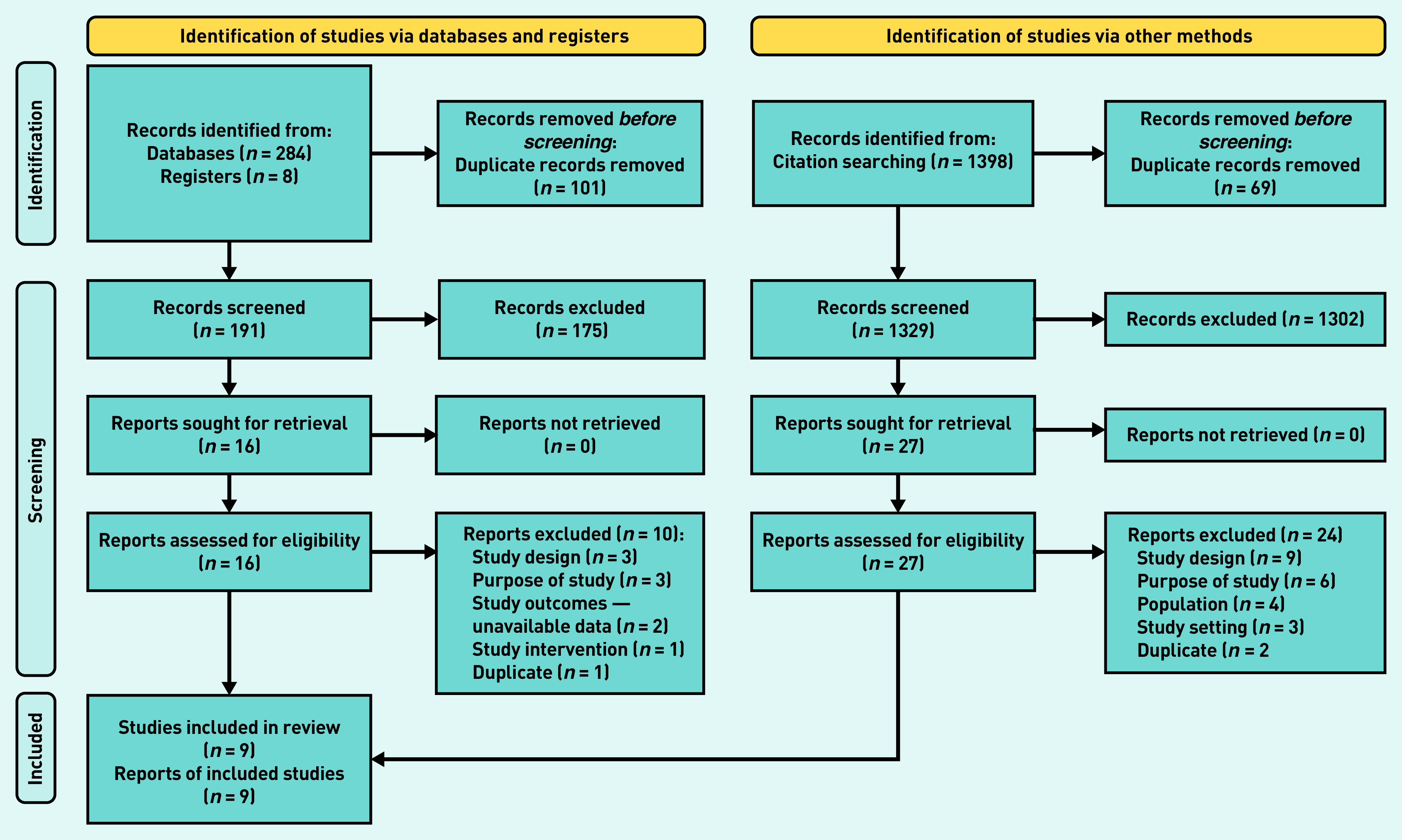
*PRISMA flowchart.^18^*

Two pairs of review authors independently screened the title and abstract of every record retrieved against the inclusion criteria to determine which studies should be assessed further. Screening was conducted using the *Screenatron* feature of the Systematic Review Accelerator.^17^^,^^19^ One author retrieved the full text and two pairs of review authors screened the full texts for inclusion. Disputes were identified using the *Disputatron* feature of the Systematic Review Accelerator and were resolved by discussion or by consulting a third author.^17^ A PRISMA flow diagram outlining the selection process (Figure 1) as well as a list of excluded full-text articles and the corresponding reasons for exclusion are provided (see Supplementary Table S1).

For studies that fulfilled the inclusion criteria, three review authors independently extracted key information on participant and intervention characteristics as well as outcomes using standard data-extraction templates. The form was piloted on two studies. Any disagreement was resolved by discussion or, if required, by a third author. Three data-extraction forms were used to collect relevant information including: table of characteristics form, primary and secondary outcomes data form, and risk of bias form (Box 1).

**Box 1.** List of extracted information

Study characteristics: country, study design, setting, and duration. Participants: sample size, age, sex, comorbidities, recent hospital admission, recent antibiotic use, and indwelling catheter. Intervention: type of antibiotic (name and class), dose, frequency, route of administration, and duration. Comparator: placebo or no treatment. Primary and secondary outcomes: development of symptomatic urinary tract infection, any-cause mortality, adverse effects of antibiotic use, antibiotic resistance, disease complications, and bacteriological cure or recurrence. Data were extracted from cohort studies on reasons for bacteriuria testing (for example, policy recommendation), when applicable.

### Risk of bias assessment

Three review authors independently assessed the risk of bias for each included RCT using the Cochrane Risk of Bias 1.0 tool.^20^ Tool 1.0 was used in preference to Tool 2.0 as the former allows the assessment of biases from conflict of interest and funding (under the ‘other sources of bias’ domain), whereas the latter does not. The following domains were assessed using the Cochrane tool:

- random sequence generation;
- allocation concealment;
- blinding (participants and personnel);
- blinding (outcome assessment);
- incomplete outcome data;
- selective reporting; and
- other sources of bias.

Each potential source of bias was graded as low, high, or unclear, and each judgement was supported by a quote from the relevant study.

Two review authors independently assessed the risk of bias for observational studies using ROBINS-I.^21^ The following domains were assessed using the ROBINS-I tool:

- bias due to confounding;
- bias in selection of participants in the study;
- bias in classification of interventions;
- bias due to deviations from intended interventions;
- bias due to missing data;
- bias in measurement of outcomes; and
- bias in selection of the reported result.

Any disagreements were resolved by discussion or by referring to a third author.

### Data synthesis

Review Manager 5.4 was used to calculate the effect of interventions.^22^ A meta-analysis was conducted where data were sufficient to pool (that is, two or more trials reporting on the same outcome). For dichotomous outcomes, risk ratios were calculated together with 95% confidence intervals (CIs). A random-effects model was used in this study, in anticipation of considerable heterogeneity. Statistical heterogeneity was assessed using the *I*^2^ statistic.

The individual was used as the unit of analysis, where possible. However, where data on the number of individuals with primary and secondary outcomes of interest were not available, the information was extracted as it was presented (for example, mean scores for the differences between groups). Investigators or study sponsors were not contacted to provide missing data.

A funnel plot was not created, as <10 studies were included in the analysis. Subgroup analyses were conducted according to duration of follow-up.

As none of the included studies were graded at ‘high’ risk of bias in three or more domains, a pre-specified sensitivity analysis assessing the impact of excluding trials rated at high risk of bias for three or more domains was not conducted.

## RESULTS

### Search results

The electronic search retrieved 284 references, supplemented with 1398 references from forward and backward citations of the included studies and eight records identified from the clinical trial registry search, resulting in 1520 records to screen after deduplication (1329 from database searches and 191 from citation searches). Screening these on title and abstract excluded 1477 references, leaving 43 articles for which full text was obtained. Screening of these full texts excluded another 34, which left nine RCTs for inclusion in this systematic review (Figure 1). Reasons for exclusions are reported in Supplementary Table S1. All clinical trials that were excluded are listed in Supplementary Table S2. The search carried out did not identify any relevant cohort studies.

### Study characteristics

Characteristics of the nine included studies are presented in Table 1. Four trials were conducted in Greece,^23^^–^26 three in the US,^27^^–^29 and two in Canada.^30^^,^^31^ Antibiotic therapies varied across trials, and included: norfloxacin, ofloxacin, tobramycin, netilmicin, trimethoprim+/-sulfamethoxazole, co-trimoxazole, ceforanide, and cefaclor. One study did not specify what antibiotic therapy was used.^27^ Two trials used a placebo comparator,^27^^,^^28^ with the remaining seven comparing against no therapy control groups. None of the identified studies investigated delayed antibiotics as a comparator arm.

**Table 1.** Characteristics of included studies^a^

**Author (year, location)**	**RCT design**	**Follow-up**	**Randomised, *n***	**Age, years, mean (SD)**	**Intervention**	**Pharmacotherapy regimen**	**Comparator: modality and dose**
Abrutyn (1994, US)^28^	Parallel quasi-RCT	9 years	358	Intervention: 81.8 Control: 82	Trimethoprim (oral) Norfloxacin (oral)	200 mg twice a day for 14 days 400 mg twice a day for 14 days	Placebo, one tablet twice a day for 14 days
Abrutyn (1996, US)^27^	Parallel quasi-RCT	9 years	358	Intervention: 82 C: 81	Not specified	Not specified	Placebo, one tablet twice a day for 14 days
Boscia (1987, US)^29^	RCT	2 years	124	Intervention: 85.8 (0.9) Control: 85.8 (0.7)	Initial therapy: trimethoprim or cefaclor (oral) Re-treatment: trimethoprim or cefaclor (oral)	Trimethoprim: 200 mg as a single dose Cefaclor: 500 mg three times a day for 3 days Trimethoprim: 200 mg twice a day for 14 days Cefaclor: 500 mg three times a day for 14 days	No therapy
Giamarellou (1998, Greece)^23^	Open-label RCT	1 year	136	Intervention 1: 84.5 (6.1) Intervention 2: 82.8 (5.2) Control: 82.9 (6.1)	Ofloxacin (oral)	Intervention 1: 200 mg twice a day for 3 days then daily for 12 weeks Intervention 2: 200 mg twice a day for 3 days, fortnightly for 12 weeks	No therapy
Giamarellou (2007, Greece)^24^	Open-label RCT	3 months	132	Intervention 1: 84.5 (6.1) Intervention 2: 82.8 (5.2) Control: 82.9 (6.1)	Ofloxacin (oral)	Intervention 1: 200 mg twice a day for 3 days then daily for 12 weeks Intervention 2: 200 mg twice a day for 3 days, fortnightly for 12 weeks	No therapy
Nicolle(1983, Canada)^30^	RCT	2 years	36	Intervention: 80.4 (12.1) Control: 80.7 (9.6)	Trimethoprim/sulfamethoxazole (oral) or tobramycin (IV) Tobramycin (IM): 1.5 mg/kg three times a day for 2 weeks	Trimethoprim/sulfamethoxazole: 160 mg/800 mg for 2 weeks	No therapy
Nicolle(1987, Canada)^31^	RCT	1 year	52	Intervention: 83.3 (8.7) Control: 83.6 (9)	Trimethoprim/sulfamethoxazole (oral) or tobramycin (IV)	Not specified	No therapy
Staszewska-Pistoni (1994, Greece)^25^	RCT	5 years	102	Intervention: 82.7 Control: 82.6	Netilmicin (IM) Co-trimoxazole (IM) Ceforanide (IM)	Netilmicin: 150 mg daily for 10 days Co-trimoxazole: 160/800 mg daily for 10 days Ceforanide: 1 g daily for 10 days	No therapy
Staszeweska-Pistoni (1995, Greece)^26^	RCT	3 months	93	Intervention 1: 84.5 Intervention 2: 82.8 Control: 82.8	Ofloxacin (oral)	200 mg twice a day for 3 days, then Intervention 1: daily for 87 days Intervention 2: 3 days every fortnight for 3 months	No therapy

a *Total number of participants randomised across the nine studies = 1391. IM = intramuscular. IV = intravenous. RCT = randomised controlled trial.*

### Risk of bias assessment

Risk of bias was generally unclear or high for random sequence generation, because of poor reporting of randomisation procedure or non-randomisation in the included trials (two of the included studies were non-RCTs), and similarly for allocation concealment. Two studies^27^^,^^28^ were rated at low risk of bias from blinding of participants and personnel, as well as outcome assessment; the remainder were rated either unclear (three studies)^25^^,^^29^^,^^30^ or high risk of bias (four studies)^23^^,^^24^^,^^26^^,^^31^ owing to the non-reporting or absence of blinding. Risk of bias because of attrition was low for most of the included studies, and risk of bias owing to selective reporting was low for all studies. The potential for other bias (arising from funding and conflict of interest issues) was mostly unclear, owing to the absence of conflict of interest and/or funding statements (see Supplementary Figures S1 and S2).

### Primary outcome: development of UTI symptoms

**Figure 2.**
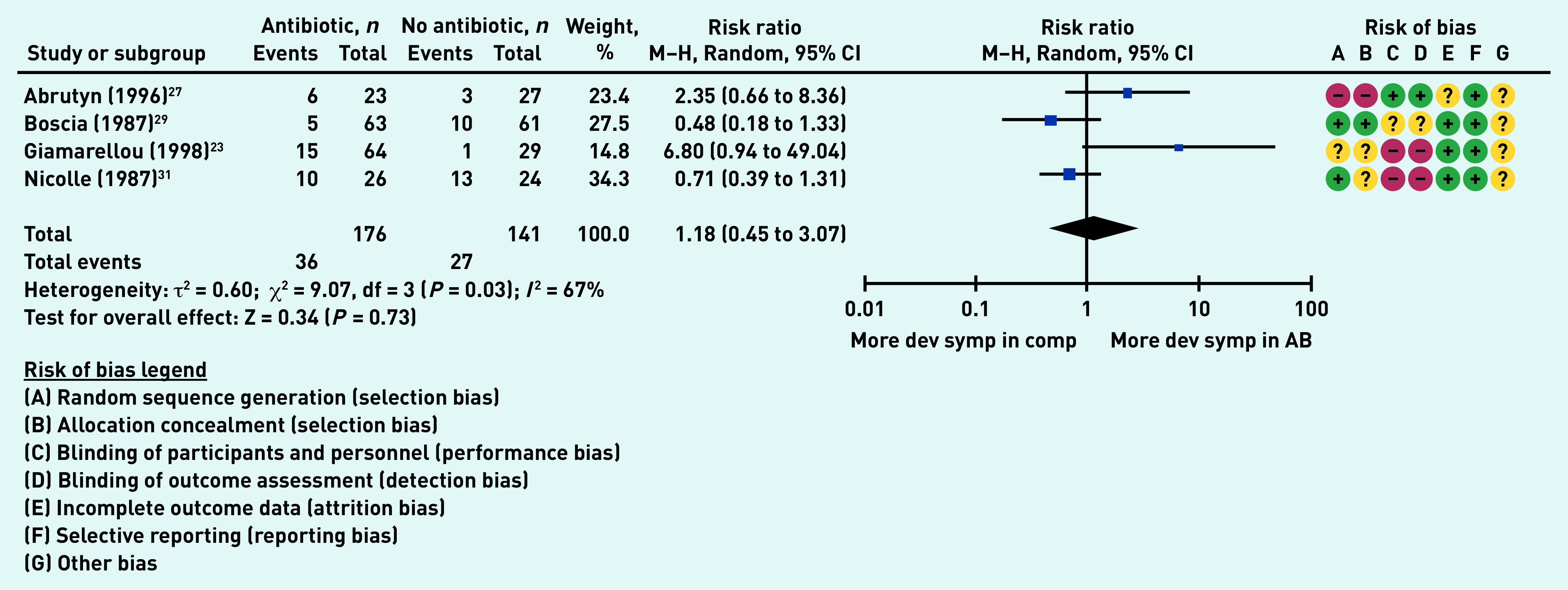
*Proportion of participants who developed urinary tract infection symptoms. AB = antibiotic group. Dev symp in comp = developed symptoms in the comparator group. df = degrees of freedom. M-H = Mantel-Haenszel.*

Four studies (317 participants in aggregate)^23^^,^^27^^,^^29^^,^^31^ reported on the number of individuals who developed a UTI. There was no difference between the antibiotic group and the comparator group (no antibiotic) (risk ratio [RR] 1.18, 95% CI = 0.45 to 3.07, *P* = 0.73).The high heterogeneity (*I* 2 = 67%) may be explained by a variety of methods used to report the outcome (for example, self-versus investigator-administered forms) (Figure 2).

### Primary outcome: adverse events

**Figure 3.**
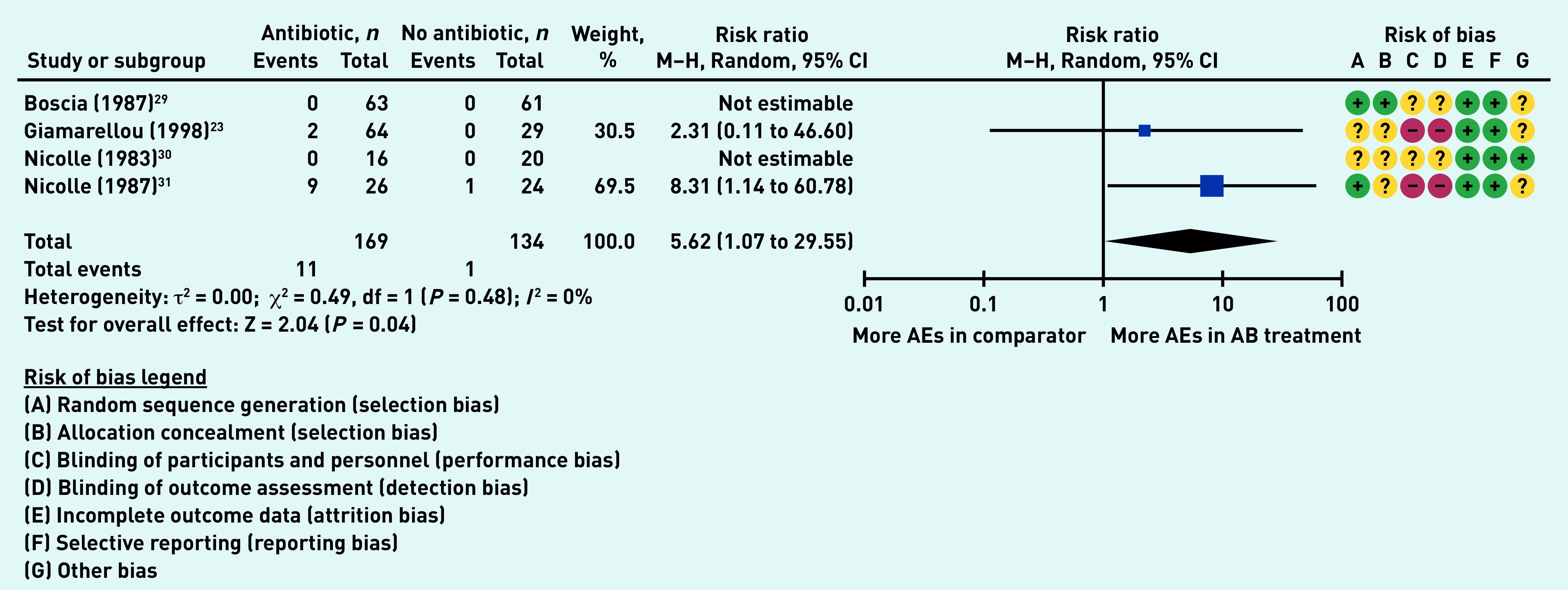
*Proportion of participants experiencing adverse events. AB = antibiotic group. AE = adverse effects. df = degrees of freedom. M–H = Mantel–Haenszel.*

Four studies (303 participants in aggregate)^23^^,^^29^^–^31 reported on the number of participants experiencing adverse events, with two studies reporting no adverse events in either the antibiotic or the no antibiotic group.^29^^,^^30^ Significantly more participants receiving antibiotics experienced adverse events (RR 5.62, 95% CI = 1.07 to 29.55, *P* = 0.04), with no heterogeneity (*I* 2 = 0%) (Figure 3).

Only Nicolle 1987 reported a breakdown of the types of adverse events experienced by participants.^31^ For the antibiotic group, these included: diarrhoea, rash, candidiasis, and swollen mouth. The corresponding comparator group (no therapy) reported only one side effect of dizziness.

### Primary outcome: mortality

**Figure 4.**
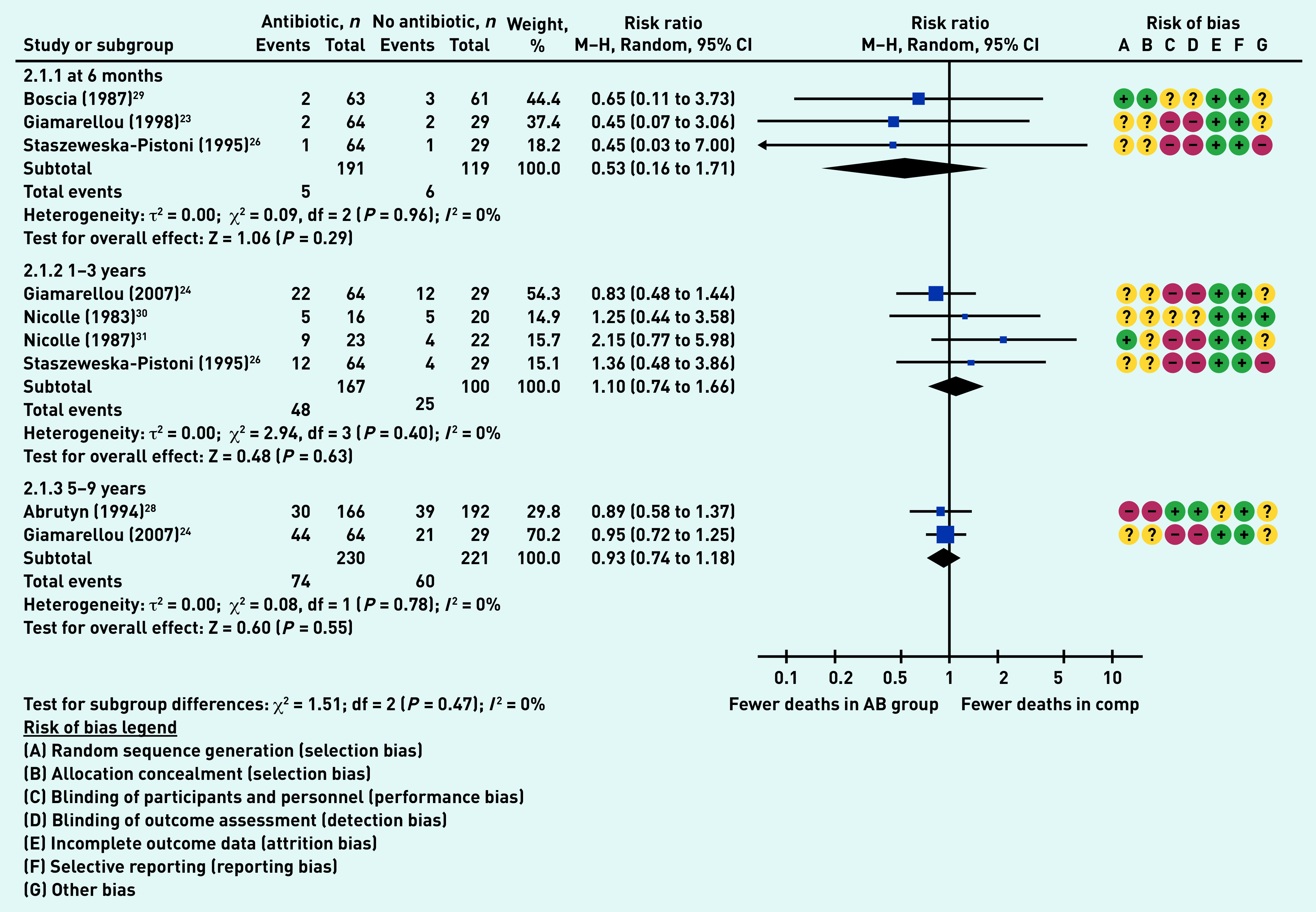
*Proportion of participants who died. AB = antibiotic group. Comp = comparator group. df = degrees of freedom. M–H = Mantel-Haenszel. 2.1.1 = mortality at 6 months. 2.1.2 = mortality at 1–3 years. 2.1.3 = mortality at 5–9 years.*

Seven studies reported on participant mortality at a variety of time points up to 9 years.^23^^,^^24^^,^^26^^,^^28^^–^31 Three studies (310 participants)^23^^,^^26^^,^^29^ reported on mortality at 6 months, with no differences between the antibiotic and comparator group (RR 0.53, 95% CI = 0.16 to 1.71, *P* = 0.29, *I* 2 = 0%). There were also no differences between groups at 1–3 years (RR 1.10, 95% CI = 0.74 to 1.66, *P* = 0.63, *I*^2^ = 0%) or at 5–9 years (RR 0.93, 95% CI = 0.74 to 1.18, *P* = 0.55, *I*^2^ = 0%) (Figure 4).

### Secondary outcome: complications

Two studies (81 participants)^30^^,^^31^ reported on complications, which included, for example, epididymo-orchitis and bacteraemia. There was no difference between groups in the number of participants experiencing complications (RR 1.89, 95% CI = 0.77 to 4.63, *P* = 0.16, *I*^2^ = 0%) (see Supplementary Figure S3).

### Secondary outcome: antibiotic resistance

Antibiotic resistance was rarely reported among the included studies, with only four studies reporting resistance of bacteria causing the infection.^23^^,^^24^^,^^30^^,^^31^ This precluded a meta-analysis. In one trial,^24^ authors reported that two-thirds of the positive urine cultures during 3 years of follow-up were resistant to ofloxacin; however, they did not report the between-group difference. In another trial,^23^ >50% of the positive urine cultures were new bacteria resistant to ofloxacin, irrespective of antibiotic exposure arm. In the third trial,^30^ authors report superinfections caused by resistant organisms; however, it was not reported which organism, and the authors did not report the between-group differences. In the fourth trial,^31^ authors reported one resident in the no therapy group with relapse/persistent infection compared with nine reported in the therapy group; however, no additional data or explanations were provided to explain the difference (see Supplementary Table S3).

### Secondary outcome: bacteriological cure

**Figure 5.**
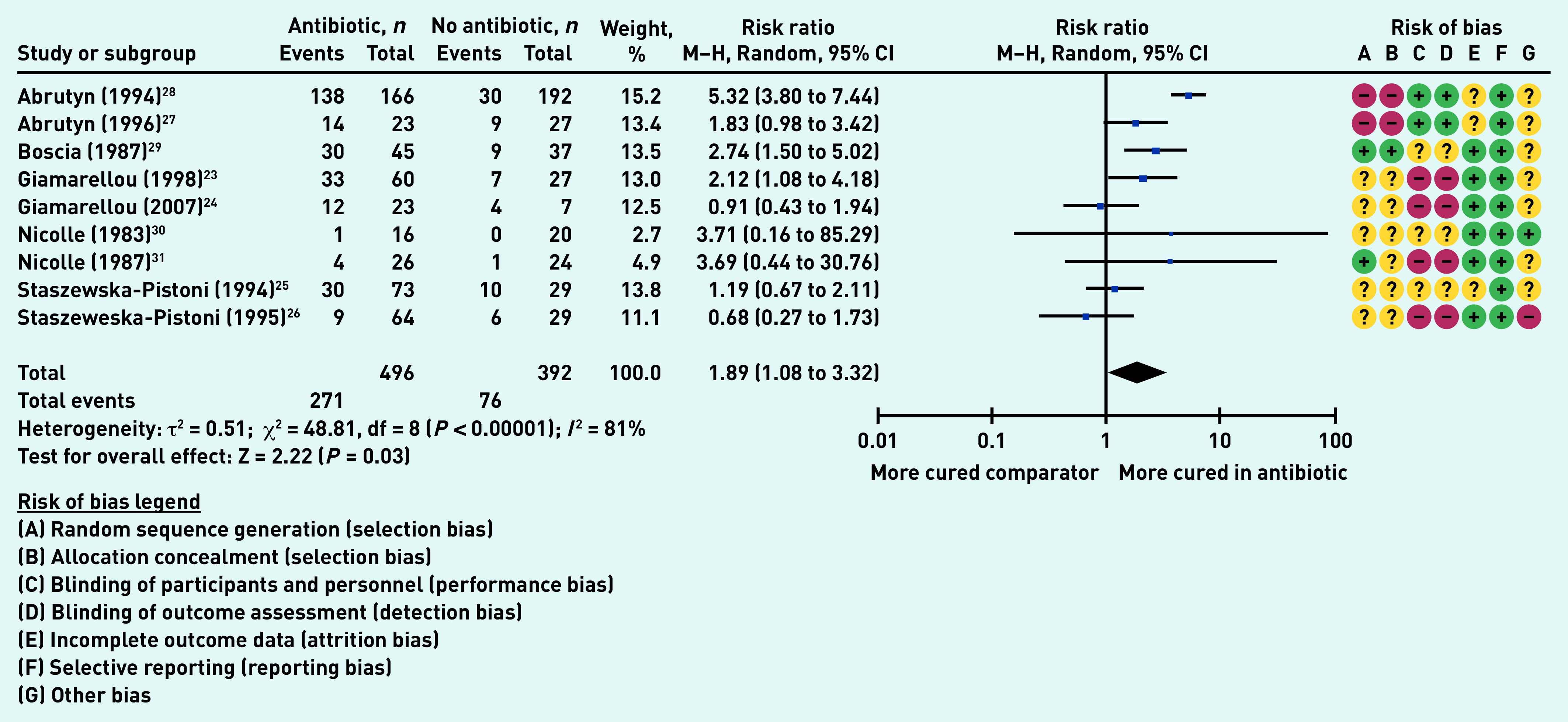
*Proportion of participants with bacteriological cure. df = degrees of freedom. M–H = Mantel–Haenszel.*

Nine studies (888 participants in aggregate)^23^^–^31 reported on the number of participants who experienced bacteriological cure. Significantly more participants in the antibiotic group than in the comparator groups experienced bacteriological cure (RR 1.89, 95% CI = 1.08 to 3.32, *P* = 0.03); however, the heterogeneity was very high (*I* 2 = 81%) (Figure 5). The high heterogeneity could be explained by the different types of antibiotic treatment, doses, and duration.

## DISCUSSION

### Summary

This systematic review of nine eligible RCTs (total of 1391 participants) suggests that antibiotic therapy compared with placebo/no therapy for older patients in RACFs was significantly more effective at achieving bacteriological cure, irrespective of developing a UTI. However, antibiotic therapy was also associated with a higher number of adverse effects, which is consistent with the findings of other reviews.^32^^,^^33^ Outcomes related to mortality, development of symptomatic bacteriuria, and complications were comparable between the two groups. This suggests that, for older patients in RACFs, the harms of antibiotic therapy for ASB may outweigh the benefits. However, these findings are based on a small number of trials and risk of bias was unclear for the majority of the domains with low-quality reporting of randomisation, allocation concealment, and blinding of participants, personnel, and outcome assessors. When considering these factors, in addition to the age of studies and the significant clinical and statistical heterogeneity, these findings should be interpreted with caution.

### Strengths and limitations

The strengths of this review lie in the search strategy, which was comprehensive, and comprised searches of three databases, trial registries, and forward and backward citation searches.

The review is also subject to some limitations. First, owing to the strict inclusion/exclusion criteria the review only included a small number of RCTs with relatively small sample sizes. Studies were limited to nursing home settings, and as such this excluded a number of trials that were based in the community (GP clinic) or hospital setting, but included participants who were based in an RACF. There was high heterogeneity across the included studies. Furthermore, there are a number of confounding variables that may affect the generalisability of results. Older patients in RACFs experience many different comorbidities, have indwelling catheters, and it is difficult to solely attribute adverse effects and mortality to antibiotic usage in this population. Furthermore, there was a lack of detail in each of the included studies outlining the diagnostic criteria used to assess ASB. This lack of information precluded comparison between studies and any conclusions being formed on the impact of diagnostic criteria differences on the results.

### Comparison with existing literature

The findings of the current review align with a Cochrane review investigating the use of antibiotics for ASB in the general adult population, across all care settings.^12^ The Cochrane review showed that there were no observed differences between antibiotics and no treatment for death, complications, or the development of symptomatic UTIs.^12^ Similarly, antibiotics were superior to no treatment for bacteriological cure and were also associated with more adverse events. The present review focuses specifically on RACF residents and includes three additional trials. Moreover, the current study expanded the search to include observational studies and non-randomised trials with a control arm.

In the context of cohort studies, a recently published retrospective cohort study, which explored antibiotic management in older patients from a general practice setting diagnosed with UTI,^34^ found that compared with antibiotic therapy, deferred or no antibiotic treatment was associated with significant increases in bloodstream infection and all-cause mortality, and as such the authors recommended first-line antibiotics for UTIs in the older population.^34^ However, it must be noted that this study assessed outcomes in a slightly different cohort of patients from the present review, as they excluded patients with ASB and focused on those with a clinical UTI diagnosis. Furthermore, the study had limitations common to observation studies using health record data, and potential biases or coding inconsistencies.

In another matched cohort study that focused on the prevention of UTIs, older adults (aged ≥66 years) receiving antibiotic prophylaxis were compared with patients who did not receive prophylaxis, and the study had similar findings to this current review.^35^ The authors found that long-term antibiotic prophylaxis was associated with higher acquisition of antibiotic resistance to any urinary antibiotic and any agent used for prophylaxis, and increased risk of hospital admission or emergency department visit because of UTI, sepsis, or bloodstream infection compared with the control group.

### Implications for research and practice

There is a need for further trials to determine the safety, effectiveness, and appropriateness of antibiotic therapy in older patients in RACFs for infections requiring antibiotic treatment. In the current review, only nine studies were identified, with <50% of the included studies reporting on three of the five usual outcomes that would need to be typically reported for this setting, age, and reported condition. This is besides the generally high or unclear risk of bias for the included studies. Future trials should aim at recruiting larger sample sizes and have clearly defined outcome criteria defining treatment failure. There is also a significant gap in knowledge relating to adverse events and antibiotic resistance data specifically in the older patient population. This could be improved by the use of standardised tools for reporting harms outcomes, such as the CONSORT harms checklist, and by the adoption of checklists specific to reporting antibiotic resistance, such as the checklist developed by several authors of the present study.^36^^,^^37^

Overall, based on nine RCTs, although antibiotic therapy was associated with bacteriological cure, it was also associated with significantly more adverse effects. The harms and lack of clinical benefit of antibiotic use for older patients in RACFs may outweigh the benefits. To provide a better indication of the effectiveness and safety of antibiotics in RACF-based patients, further primary studies are warranted.

## Data

The systematic review protocol is available at Open Science Framework: https://osf.io/f8uka. Data extraction forms are available from the corresponding author. The complete search strategy is available in Supplementary Box S1. Reasons for exclusions and excluded clinical trials are reported in Supplementary Table S1 and Table S2, respectively. The complete risk of bias assessment is available in Supplementary Figures S1 and S2.
